# Carbon Monoxide Poisoning:From Microbes to Therapeutics

**DOI:** 10.1146/annurev-med-052422-020045

**Published:** 2023-08-15

**Authors:** Matthew R. Dent, Jason J. Rose, Jesús Tejero, Mark T. Gladwin

**Affiliations:** 1 Heart, Lung, Blood and Vascular Medicine Institute, University of Pittsburgh, Pittsburgh, Pennsylvania, USA; 2 Department of Medicine, University of Maryland School of Medicine, Baltimore, Maryland, USA; 3 Division of Pulmonary, Allergy and Critical Care Medicine, University of Pittsburgh, Pittsburgh, Pennsylvania, USA; 4 Department of Bioengineering, Swanson School of Engineering, University of Pittsburgh, Pittsburgh, Pennsylvania, USA; 5 Department of Pharmacology and Chemical Biology, University of Pittsburgh, Pittsburgh, Pennsylvania, USA

**Keywords:** carbon monoxide poisoning, carbon monoxide, antidotes, mitochondria, heme

## Abstract

Carbon monoxide (CO) poisoning leads to 50,000–100,000 emergency room visits and 1,500–2,000 deaths each year in the United States alone. Even with treatment, survivors often suffer from long-term cardiac and neurocognitive deficits, highlighting a clear unmet medical need for novel therapeutic strategies that reduce morbidity and mortality associated with CO poisoning. This review examines the prevalence and impact of CO poisoning and pathophysiology in humans and highlights recent advances in therapeutic strategies that accelerate CO clearance and mitigate toxicity. We focus on recent developments of high-affinity molecules that take advantage of the uniquely strong interaction between CO and heme to selectively bind and sequester CO in preclinical models. These scavengers, which employ heme-binding scaffolds ranging from organic small molecules to hemoproteins derived from humans and potentially even microorganisms, show promise as field-deployable antidotes that may rapidly accelerate CO clearance and improve outcomes for survivors of acute CO poisoning.

## INTRODUCTION

Carbon monoxide (CO) presents an intriguing dichotomy in human health and disease. On the one hand, exposure to CO, largely derived from the incomplete combustion of carbon-based fuels, is responsible for 50,000–100,000 emergency room visits and 1,500–2,000 deaths each year in the United States alone (1). On the other hand, CO, which is produced endogenously as a product of heme degradation by heme oxygenases (2), is implicated in biological signaling cascades that regulate inflammation, cell growth/proliferation, and circadian rhythm (3, 4). Preclinical and clinical data even suggest that exposure to low concentrations of gaseous CO or synthetic CO-releasing molecules (CORMs) mitigates cellular damage in models of sepsis, ischemia/reperfusion injury, and organ transplantation (5–7). The dual nature of CO is also present in the microbial world, where CO acts as a toxin at high concentrations but also serves as a nutrient for specialized CO-metabolizing microbes found in diverse environments ranging from deep sea hydrothermal vents to the human gut (8, 9). In this review, we provide an overview of the prevalence and impact of CO poisoning and its pathophysiology in humans. We also highlight recent advances in targeted CO antidotal therapies, focusing on promising efforts to develop high-affinity CO sequestration agents and the potential utility of microbial CO sensor proteins as scaffolds to design new CO scavengers.

## IMPACT OF CARBON MONOXIDE POISONING AND UNMET MEDICAL NEED

### Prevalence and Long-Term Ramifications of CO Poisoning

CO poisoning remains the most common cause of non–drug related human poisoning and a major cause of death and disability (1, 10). CO is produced at toxic levels by the incomplete combustion of carbon-based fuels from anthropogenic sources such as engine exhaust, fires, and gasoline-powered tools. The acute clinical effects of severe CO poisoning include cognitive dysfunction that can progress to brain injury and coma, cardiovascular complications, and death (11, 12). Carboxyhemoglobin (HbCO) levels greater than 25% typically lead to systemic acidosis (pH < 7.2), loss of consciousness, and need for endotracheal intubation (13). Up to half of the patients with moderate to severe CO poisoning (i.e., those admitted to the hospital or critically ill) present with myocardial injury or left ventricular dysfunction (12, 14, 15).

Survivors of CO poisoning have increased long-term mortality compared to nonpoisoned, age-matched patients, particularly in cases of intentional poisonings (16). In a study of patients hospitalized for CO poisoning with a median follow-up time of 7.6 years, the mortality rate was 38% in survivors who suffered myocardial injury versus 15% for survivors without cardiac complications. Myocardial injury has been shown to be an independent predictor for short-term mortality and neurocognitive sequelae (17, 18), and survivors are also at additional risk of future myocardial infarction (14). All survivors of CO poisoning are at risk of long-term neurocognitive sequelae, which often present in the days and weeks following initial CO poisoning (19–22). In long-term follow up studies, up to 40% of patients develop neurological or cognitive disturbances (10, 23–25). Additionally, CO poisoning victims report significant levels of posttraumatic stress disorder and depression as well as lower quality of life (26). The main causes of long-term mortality in survivors of CO poisoning are alcoholism, motor vehicle accidents, other accidents, and intentional self-harm, all of which are possible sequelae of neuropsychiatric dysfunction. These causes may also be associated with prior mental illness and/or high-risk behavior, particularly in the case of intentional CO poisoning (26). Current guidelines recommend following up within 1–2 months of poisoning to assess the development of neurological and cognitive deficits, depression, and/or anxiety (10). Unfortunately, there are no current treatments for patients with long-term neurocognitive sequelae from CO poisoning.

### Current Therapeutic Options and Limitations

There is no point-of-care antidotal therapy available for CO poisoning. Current therapeutic options are mainly limited to normobaric oxygen therapy (NBOT) and hyperbaric oxygen therapy (HBOT). Patients exposed to CO sources are typically treated with normobaric (1 atm) 100% oxygen (O_2_) to increase intrinsic CO clearance through the lung alveoli. If possible, these patients are transferred to a facility with a hyperbaric (1.5–3 atm) 100% O_2_ delivery system. The half-life of CO in the human circulation is ∼320 min in room air but only 74 min with NBOT and 20 min with HBOT (1). In a meta-analysis of all clinical trials comparing O_2_ delivery to treat CO poisoning, no significant reduction in long-term neurocognitive sequelae was observed for patients treated with HBOT compared to NBOT (27). The single trial that met all Consolidated Standards of Reporting Trials (CONSORT) criteria and measured one-year outcomes did show significant benefit in long-term neurocognitive dysfunction with HBOT treatment (28, 29). While HBOT may be more effective than NBOT, this treatment option is only available at ∼350 centers in the United States. At those centers, hyperbaric chambers have a standard time of access post diagnosis of 5 h (28), by which time blood HbCO levels are low even with the application of NBOT. Moreover, the studies that have shown HBOT to be beneficial suggest that neurological impairment, while improved, still occurs in up to 40% of survivors of CO poisoning who receive this treatment (1, 10, 21, 28).

A sequential gas delivery apparatus that induces isocapnic hyperpnea was recently approved by the US Food and Drug Administration. This device is utilized in conjunction with NBOT to accelerate CO clearance by increasing minute ventilation through the administration of carbon dioxide (CO_2_) gas (30, 31). In a study of 13 healthy, chronically smoking volunteers, NBOT with isocapnic hyperpnea accelerated CO clearance in circulation [*t*_1/2_(HbCO) = 29.6 ± 12.2 min] compared to NBOT alone [(*t*_1/2_(HbCO) = 47.3 ± 19.2 min] (32). Isocapnic hyperpnea has not been studied for clinical outcomes beyond CO clearance, and there remains hesitance over administering inhaled CO_2_ gas to potentially obtunded patients with depressed respiratory function. Given the limitations of currently approved treatments, there is great need for a point-of-care therapy to treat CO poisoning in the emergency room or the field.

## CURRENT UNDERSTANDING OF CARBON MONOXIDE POISONING PATHOPHYSIOLOGY IN MAMMALS

### CO Binding to Biological Hemoprotein Targets

**Figure 1:**
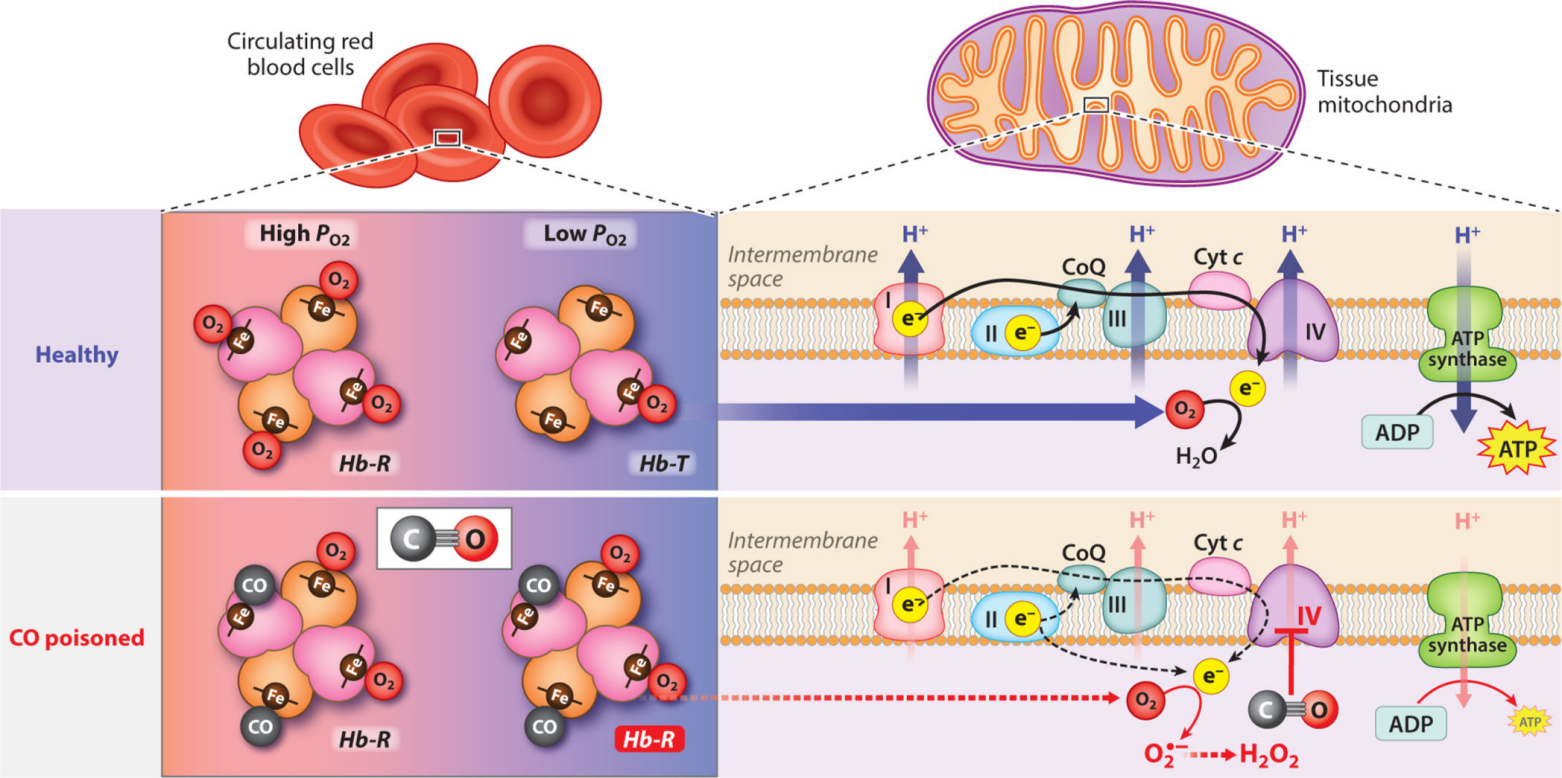
Pathophysiological effects of acute CO exposure. In circulating red blood cells, CO binds to heme sites in Hb, diminishing O_2_ carrying capacity and stabilizing the high-affinity R-state. In addition to binding Hb in circulation, CO may also bind myoglobin, causing a reduction in O_2_ availability in tissue. In mitochondria, CO may directly bind the heme *a*_3_ site in complex IV of the mitochondrial ETC, inhibiting the reduction of O_2_ to water. This inhibition, coupled with reduced O_2_ availability, leads to membrane depolarization, reduced ATP output, and accumulation of reducing equivalents in the ETC. These reducing equivalents may react with O_2_ to form superoxide (O_2_*•*−), which may propagate cellular damage directly or through conversion to other reactive oxygen species, such as hydrogen peroxide (H_2_O_2_). Abbreviations: CoQ, coenzyme Q/ubiquinone; Cyt *c*, cytochrome *c*; ETC, electron transport chain; Hb-R, relaxed-state hemoglobin; Hb-T, tense-state hemoglobin; *P*_O2_, partial pressure of O_2_. Figure adapted from images created with BioRender.com.

At the molecular level, CO affects biological systems through binding interactions with the ubiquitous iron-containing cofactor heme, and more than 20 distinct mammalian hemoprotein targets of CO have been characterized to date (3). The consequences of CO binding to these hemoproteins are numerous and vary greatly depending upon physiological context. Central to the pathophysiology of acute CO poisoning are binding interactions between CO and two heme sites: hemoglobin (Hb) and the heme *a*_3_ site of cytochrome *c* oxidase (COX or complex IV) in the mitochondrial electron transport chain (Figure 1).

CO binding to Hb limits O_2_ carrying capacity and delivery in circulation. Hb exhibits between 200- and 400-fold higher affinity for CO compared to O_2_ (Table 1) (33, 34). During CO inhalation in the lung, CO outcompetes O_2_ for heme binding sites and diminishes the O_2_ carrying capacity of Hb. As with O_2_, CO binding to Hb facilitates an allosteric transition from low-affinity, tense-state (Hb-T) to high-affinity, relaxed-state (Hb-R) (35). This allosteric effect further increases ligand binding affinity, shifting the oxy-hemoglobin dissociation curve to the left and thereby reducing the ability of Hb to release O_2_ in peripheral tissues where O_2_ partial pressures (*P*_O2_) are low (36). These two effects, reduced O_2_ carrying capacity and stabilization of R-state Hb, limit O_2_ delivery to tissues during acute CO poisoning.

Due to the dynamic nature of CO binding to Hb, inhaled CO that binds Hb in the pulmonary vasculature may dissociate from heme and diffuse to other tissues. In cardiac and skeletal muscle, myoglobin (Mb), which exhibits a nanomolar binding dissociation constant for CO (*K*_d,CO_ = 37 nM), represents an abundant potential target for CO and a potential CO reservoir (Table 1) (37). CO binding to Mb may thus further impair delivery of O_2_ in these tissues. CO binding has been described for two additional mammalian globin proteins, neuroglobin (Ngb) and cytoglobin (Cygb) (38, 39). While the precise biological functions of these proteins remain incompletely understood, high ligand binding affinities suggest that Ngb and Cygb do not act as strict O_2_ carriers in tissue (Table 1) (40). Ngb in particular exhibits a remarkably high CO binding affinity (*K*_d,CO_ = 0.2 nM) (38), and CO bound to Ngb in the brain has been suggested to act as a long-term CO reservoir that contributes to prolonged neurological damage after CO exposure (41).

The heme *a*_3_ site of COX represents a second important biological target of CO during acute CO poisoning (Figure 1). CO binds to this heme site with moderate binding affinity (*K*_d,CO_ = 0.32 μM, Table 1) (42, 43), acting as a competitive inhibitor for the O_2_ substrate (*K*_d,O2_ = 0.1 μM) that is typically converted to water by reducing equivalents generated in the mitochondrial electron transport chain. Inhibition of COX by CO attenuates the respiratory capacity of mitochondria, resulting in decreased ATP production in tissues (44, 45). A buildup of reducing equivalents in the electron transport chain results in formation of superoxide and other reactive oxygen species (ROS), which may cause oxidative damage and trigger activation of downstream inflammatory and apoptotic pathways (46, 47). Resumption of O_2_ delivery to tissues following CO clearance after acute CO poisoning should restore COX activity and ATP production, although, depending on the time to restoration and extent of ischemia-reperfusion injury, the damage from ROS and ATP loss may be responsible for myriad long-term symptoms (46, 48).

### Downstream Pathophysiological Effects of Acute CO Poisoning

Clinical case reports and animal studies reveal prolonged attenuation of COX activity following acute CO exposure even after HbCO levels have returned to baseline (45, 49). Such prolonged impairment of COX activity is consistent with the long-term neurocognitive and cardiac complications associated with acute CO poisoning, which often manifest in the days and weeks following exposure and subsequent treatment (50). Several pathophysiological mechanisms link prolonged impairment of COX activity and long-term sequelae following CO poisoning. First, CO off-loaded in peripheral tissues during acute CO exposure may tightly bind other high-affinity hemoprotein targets and slowly release CO over the course of hours or days following clinical resolution (restoration of HbCO to basal levels). Second, mitochondrial damage incurred during poisoning may increase the amount of labile heme, leading to sustained generation of CO through the action of the inducible heme-degrading heme oxygenase 1 (HO-1) (51). Third, as mentioned, oxidative species generated by COX inhibition during CO exposure may trigger signal cascades that alter physiological function on a longer timescale (46).

Through direct cellular damage and downstream signal cascades, ROS generated during acute CO poisoning may contribute to long-term neurological and cardiac sequelae. Apoptosis, ischemia-reperfusion injury, and unchecked immune response are likely driven by ROS signaling, and several recent reviews summarize efforts to characterize these pathophysiological mechanisms (1, 46, 48). In short, ROS generated during CO exposure can overwhelm cellular redox buffering systems (52), facilitate excessive lipid peroxidation (often mediated by reactive nitrogen species such as peroxynitrite) (53–56), trigger neutrophil degranulation and adhesion (57, 58), and cause release of excitatory amino acids (59). In addition to mitochondria, ROS derived from cellular NADPH (nicotinamide adenine dinucleotide phosphate) oxidases and xanthine oxidase are key sources of cellular damage in the context of acute CO poisoning (60–62). The consequences of ROS-mediated damage are thought to be particularly severe in the brain, where lipid oxidation products conjugate myelin basic protein, forming an adduct that activates microglia and promotes further immune response, which leads to neurodegeneration (63).

## NONPHARMACOLOGICAL GAS EXCHANGE TO TREAT CARBON MONOXIDE POISONING

**Figure 2:**
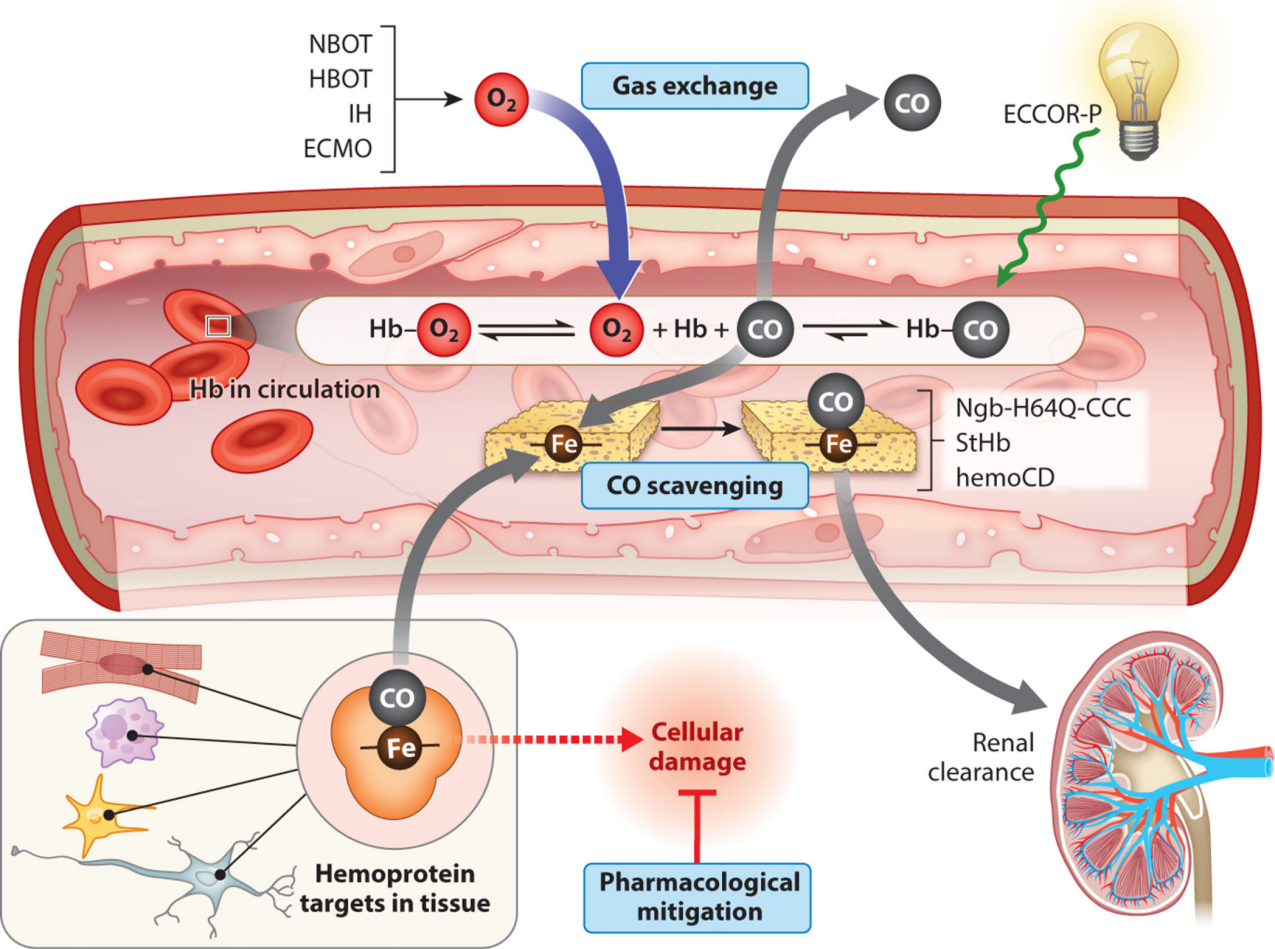
Summary of treatment strategies for acute CO poisoning. Treatment strategies fall into three categories: gas exchange, CO scavenging, and pharmacological mitigation. Normobaric oxygen therapy (NBOT), hyperbaric oxygen therapy (HBOT), and isocapnic hyperpnea (IH) therapy rely on gas exchange in the lungs with accelerated CO clearance driven by increased O_2_ partial pressure or ventilation. Extracorporeal membrane oxygenation (ECMO) may facilitate CO clearance via gas exchange in cases of extreme illness or lung damage, and extracorporeal removal of CO with phototherapy (ECCOR-P) facilitates this clearance through CO photolysis. CO-scavenging therapeutics, including hemoprotein-based scavengers (Ngb-H64Q-CCC, StHb) and small molecule–based scavengers (hemoCD), may directly sequester CO from circulating hemoglobin (Hb) or cellular hemoproteins. These scavengers undergo rapid renal clearance to safely eliminate both scavenger and CO. A number of pharmacological compounds have been explored to mitigate the downstream pathophysiological damage incurred during acute CO poisoning, including steroids, anti-inflammatory drugs, and mitochondrial electron transport chain substrates. Figure adapted from images created with BioRender.com.

Several strategies have been explored in preclinical models and in clinical settings to accelerate CO clearance during acute CO poisoning (Figure 2). Two case studies in humans (one adult study and one pediatric) with severe CO poisoning have demonstrated accelerated CO clearance and patient survival upon extracorporeal membrane oxygenation (ECMO) (64–66), although no large, randomized clinical trials have been conducted to fully assess safety, efficacy, or improvement of long-term outcomes (67). Recognizing the photosensitive nature of the bonding interaction between heme iron and CO, Zazzeron et al. have recently combined ECMO with CO phototherapy to further enhance clearance of CO from circulation in preclinical rodent models of CO poisoning (68, 69).

## PHARMACOLOGICAL MITIGATION OF CARBON MONOXIDE POISONING EFFECTS

While still not fully understood, the pathophysiological mechanisms that propagate cellular damage induced by CO poisoning provide additional downstream therapeutic targets to mitigate long-term sequelae (1). Steroids and other anti-inflammatory drugs have been tested in rodents to mitigate immune response (70), and the xanthine oxidase inhibitor allopurinol reduces neurological damage in CO poisoned rats (60). Some therapies focused on restoring mitochondrial function, either through ROS scavenging or supplementation with cell-permeable succinate, may serve as promising downstream therapeutic routes to treat long-term tissue damage associated with CO poisoning (71). The injection of intravenous hydroxycobalamin with ascorbic acid has been studied in rat models of CO poisoning. This treatment appears to improve the clearance rate of CO, apparently by promoting CO oxidation to CO_2_ that is easily cleared during respiration (72). A possibility not considered in this study is that ascorbic acid coinfused with hydroxycobalamin also acts as a radical scavenger and mitigates cellular damage.

## PHARMACOLOGICAL CARBON MONOXIDE SEQUESTRATION

### Principles of CO Sequestration

CO interacts with Hb via a dynamic equilibrium in which CO molecules rapidly associate and dissociate at Hb heme sites. A pharmaceutical CO scavenger present in circulation could serve as a CO sink that sequesters off-loaded CO before it can rebind Hb or escape circulation to poison the surrounding tissue (Figure 2). Our research group has characterized the ideal properties of an effective CO scavenging antidote. Such a scavenger must exhibit high CO binding affinity (*K*_d_ values in the low nanomolar to picomolar range) to outcompete hemoprotein targets in the body and accelerate CO clearance from biological tissue (41, 73). Importantly, a scavenger must also exhibit selectivity for CO over other diatomic molecules, particularly O_2_, which is abundant in circulation and would otherwise compete for scavenger binding. A succinct expression for CO selectivity over O_2_, the *M*-value, is computed as a ratio of binding association equilibrium constants for CO and O_2_ (*M* = *K*_a,CO_/*K*_a,O2_, Table 1). The ideal scavenger will remain tightly bound to CO even after renal excretion, allowing for safe elimination in the urine. If suitable for deployment in the field and in emergency departments, such a pharmaceutical scavenger could provide rapid acceleration of CO clearance without the delays and specialty equipment needed for HBOT (28). Given the strong interaction between CO and heme, our lab and others have employed heme, bound to a protein or small-molecule scaffold, in the design of pharmaceutical CO scavengers with therapeutic potential (41, 74–77).

To maximize safety and efficacy, a heme-based CO scavenger must meet several additional criteria beyond favorable ligand binding properties. The heme iron must be stable in the ferrous (Fe^2+^) oxidation state and exhibit slow autoxidation [conversion of oxyferrous heme to ferric (Fe^3+^) heme plus superoxide], as ferric heme does not bind CO. The scaffold must exhibit thermal and chemical stability to prevent the release of free heme, which itself may cause oxidative stress (78). Finally, the intact scavenger (scaffold plus heme) must be inert to adverse reactivity (e.g., oxidant generation) under physiological conditions.

### Hemoprotein-Based CO Scavengers

This class of CO scavengers relies on a protein polypeptide chain to act as a heme-binding scaffold and offers several distinct advantages. The design of these scavengers may draw on well-characterized hemoproteins from the literature. As a result, hemoprotein design need not be a de novo process, and proteins bearing promising properties can be used as scaffolds. These scaffolds can be further optimized by altering the native amino acid sequence using mutagenesis. On the other hand, hemoprotein-based scavengers tend to be larger (at least 15 kDa) than small-molecule reagents (reported as small as 4 kDa), which results in larger mass doses and slower renal elimination (79, 80). Production of cofactor-loaded hemoprotein at a large scale represents another potential challenge in the wider implementation of these scavengers in the clinic.

Our lab first demonstrated the utility of a pharmaceutical CO scavenger in treating acute inhaled CO poisoning in a murine model (74). In this proof-of-concept study, an engineered variant of human neuroglobin (Ngb-H64Q-CCC) was employed as a high-affinity, hemoprotein-based CO scavenger. Unlike Hb and Mb, native Ngb bears a 6-coordinate heme in which the distal His64 directly binds to the heme iron. Replacement of His64 with a noncoordinating Gln residue (H64Q) results in a five-coordinate heme with picomolar affinity for CO (*K*_d,CO_ = 2.6 pM) and almost 10,000-fold higher selectivity for CO over O_2_ (74).

Ngb-H64Q-CCC effectively sequesters CO from red blood cells, attenuates CO-dependent inhibition of COX, and improves survival and hemodynamic outcomes in murine models of acute inhaled CO poisoning. In a lethal model of CO poisoning, mechanically ventilated mice exposed to 30,000 ppm CO for 4 min experienced bradycardia, hypotension, and death if left untreated (74). Treatment with a single dose of oxyferrous Ngb-H64Q-CCC reversed hemodynamic collapse and significantly improved survival compared to control animals. Improved physiological outcomes were accompanied by accelerated clearance of CO from circulation and reduced lactate levels (74). Circulating Ngb-H64Q-CCC in plasma was completely CO saturated by the end of infusion, consistent with rapid CO scavenging kinetics. In a follow-up study, improved respiration was observed in heart tissue from CO-poisoned mice treated with Ngb-H64Q-CCC compared to control animals (81). Further, isolated mitochondria exposed to CO in vitro exhibited improved COX activity upon treatment with Ngb-H64Q-CCC (81).

Despite the efficacy of Ngb-H64Q-CCC in treating acute CO poisoning in murine models, there remain translational challenges for broad clinical use. In a screen for organ-specific toxicity in mice, oxyferrous Ngb-H64Q-CCC administered after CO poisoning underwent rapid renal clearance with minimal toxic effects as measured by organ histology and blood chemistry (74). However, ferric Ngb-H64Q-CCC administered to healthy (non-CO-poisoned) mice produced kidney damage. This discrepancy in toxicity likely arises from the fact that 5-coordinate, ferric Ngb reacts with hydrogen peroxide (abundant in the kidney) to form highly reactive heme compounds capable of causing oxidative damage (82).

Based on the promising proof-of-concept results with Ngb, our lab has investigated if other hemoproteins, more readily available in large quantities, exhibit utility as CO-scavenging agents. In a recent study by Xu et al., two strategies that stabilize the high-affinity R-state were employed using Hb from expired, leukoreduced human donor packed red blood cells (75). First, 2,3-diphosphoglycerate, which stabilizes the low-affinity T-state (83), was stripped from Hb (StHb). Second, Cys93 of the β-Hb subunit was reacted with N-ethylmaleimide (NEM-Hb) to irreversibly lock the protein in the R-state conformation (84, 85). While StHb and NEM-Hb CO binding affinities are moderate (Table 1), both modified Hb species improved survival to the same extent as Ngb-H64Q-CCC in the same lethal CO poisoning model at similar doses (75). In contrast to Ngb-H64Q-CCC, only two-thirds of heme sites in StHb and NEM-Hb bind CO upon infusion. The remainder of heme sites are free to bind and deliver O_2_, possibly providing an additional pharmaceutical benefit. Unlike Ngb-H64Q-CCC, StHb and NEM-Hb do not elicit organ-specific toxicity upon infusion in healthy mice. Further, scavenger-induced hypertension due to NO scavenging, a primary safety concern of cell-free Hb (86), was not observed for StHb or NEM-Hb in the context of CO poisoning, although hypotensive effects in the absence of CO were not investigated.

### Small Molecule–Based CO Scavengers

The heme cofactor is inherently hydrophobic and therefore sparingly soluble in water. Moreover, free heme can cause cellular damage through oxidant-generating Fenton-type reactions and through stimulation of immunogenic response via TLR-4 activation (78). While nature has solved these problems by burying heme in the hydrophobic cores of proteins, small-molecule biomimetic heme compounds often suffer from poor solubility in water and rapid auto-oxidation in air (87). Despite these chemical challenges, a distinct advantage of small-molecule scavengers is their small size, allowing for lower-mass doses and very fast renal clearance.

In a series of papers, Kitagishi and colleagues developed and characterized a set of water-soluble porphyrins that exhibit promising CO-scavenging properties (88–90). In these small-molecule scavengers, two amphipathic β-cyclodextrin (CD) rings are covalently bridged by a metal-coordinating linker moiety (91). The hydrophobic cores of the CD rings cap an iron-bound, meso-substituted porphyrin (FeTPPS) while the linker directly coordinates to the metal center (92), forming a five-coordinate, hemoprotein-like species (hemoCD). The best characterized of these compounds, which bears a pyridine linker moiety (hemoCD-P), exhibits good solubility (∼15 mM in aqueous buffer), picomolar affinity for CO (*K*_d,CO_ = 19.2 pM), high selectivity for CO over O_2_ (*M* = 1.4 × 10^6^), and slow autoxidation (*k*_autox_ = 0.14 h^−1^ at 37°C) (77, 89, 90).

In the context of acute CO poisoning, hemoCD-P has recently been employed as a scavenger and a colorimetric tool to assess tissue CO accumulation. By treating tissue homogenates with hemoCD-P, Mao et al. developed a method to quantify tissue accumulation of CO in rat models of acute inhaled CO poisoning using spectrophotometry (41). This study revealed that CO persists in many different tissues (lung, brain, heart, and skeletal muscle) even after 1 h treatment with 100% O_2_. Combined treatment with hemoCD-P and O_2_ inhalation accelerated clearance of CO from brain tissue compared to O_2_ inhalation alone, although no acceleration of clearance was observed for heart, liver, lung, or skeletal muscle (41). Mao et al. recently demonstrated that a combination of hemoCD-P and another imidazole-bridged compound (hemoCD-I), dubbed hemoCD-Twins, effectively treats dual CO and cyanide (CN^−^) poisoning in rodent models, a common “dual-hit” smoke inhalation injury observed in victims of fire accidents (77). Administration of hemoCD-Twins at a therapeutic dose of 56 mg/kg did not elicit organ-specific toxic effects after 24 h in healthy (non-CO-poisoned) mice, suggesting preliminary safety of this compound (77).

### CO Sequestration Strategies from the Microbial World

Given the open questions regarding safety, efficacy, and pharmacological implementation strategies for heme-based CO scavengers, alternative molecules should be considered. To that end, our lab has recently turned to the world of CO-metabolizing microbes, a diverse class of organisms that utilize environmental CO as a source of carbon and/or cellular energy (8, 9). These organisms, which are found in a variety of anoxic and O_2_-replete environments, employ CO dehydrogenase (CODH) enzymes to catalyze the reversible two-electron oxidation of CO to CO_2_. The use of CODH enzymes could provide a means of catalytic CO removal in the context of acute CO poisoning; however, these enzymes are composed of multimeric enzyme complexes with numerous metal-containing cofactors, presenting significant challenges for recombinant expression and long-term stability (93).

The production of oxidative CO metabolism genes incurs a high energetic cost for cells, and CODH expression is therefore tightly regulated at the transcriptional level by heme-dependent, CO-sensing transcription factors (94). One of these transcription factors, initially isolated from the aerobic soil bacterium *Paraburkholderia xenovorans*, is the regulator of CO metabolism (RcoM) protein (95). CO binding to ferrous heme activates RcoM DNA binding function and subsequent CODH expression (96, 97). The native RcoM protein binds CO with nanomolar binding affinity (*K*_d_ = 4 nM), and CO binding is further enhanced in a truncated version of the protein bearing the heme-binding domain alone (*K*_d_ = 0.25 nM) (98). Importantly, the CO-sensing function of native RcoM is carried out under aerobic conditions, suggesting that this protein may possess selectivity for CO over O_2_ in addition to its remarkably high CO-binding affinity. Given these promising ligand binding properties, our lab is actively pursuing the development of RcoM as a hemoprotein-based CO scavenger (99).

## CONCLUSIONS

While not completely understood, the pathophysiology of acute CO poisoning clearly originates with CO–heme binding interactions in circulating Hb and at heme sites in tissue. Numerous experimental therapeutic strategies have been explored to improve survival outcomes and mitigate long-term sequelae in patients who experience acute CO poisoning primarily through methods that accelerate gas exchange or mitigate downstream pathophysiological signaling pathways. Despite these efforts, a comprehensive treatment strategy remains elusive. Pharmacological CO scavengers hold great potential as therapeutic agents to treat acute CO poisoning. Unlike HBOT, which requires specialized facilities and patient transport, a pharmacological CO-scavenging therapeutic may be deployed directly in the field, clinic, or emergency department, allowing for rapid acceleration of CO clearance. In recent years, several heme-based therapeutics have demonstrated CO-scavenging efficacy with improved physiological outcomes in rodent models of acute CO poisoning. These exciting proof-of-concept studies should fuel further work aimed at optimizing CO-scavenging properties for maximal efficacy and safety, in addition to assessing long-term benefits of scavenger treatment in the context of acute CO poisoning.

## Figures and Tables

**Table 1:** Summary of ligand binding parameters for select physiological targets of CO and potential CO scavenging therapeutics

Protein	O_2_				CO				*M*-value	References
	*k*_on_ (M^−1^s^−1^)	*k*_off_ (s^−1^)	*K*_a_ (M^−1^)	*K*_d_ (M)	*k*_on_ (M^−1^s^−1^)	*k*_off_ (s^−1^)	*K*_a_ (M^−1^)	*K*_d_ (M)		
Hb-T	4.50 × 10^6^	1900	2.37 × 10^3^	4.22 × 10^−4^	8.30 × 10^4^	0.09	9.22 × 10^5^	1.08 × 10^−6^	389	33
Hb-R	5.00 × 10^7^	15	3.33 × 10^6^	3.00 × 10^−7^	6.00 × 10^6^	0.01	6.00 × 10^8^	1.67 × 10^−9^	180	33
Mb	1.40 × 10^7^	12	1.17 × 10^6^	8.57 × 10^−7^	5.10 × 10^5^	1.90 × 10^−2^	2.68 × 10^7^	3.73 × 10^−8^	23	37
Heme *a*_3_ (COX)	1.00 × 10^8^	10	1.00 × 10^7^	1.00 × 10^−7^	7.20 × 10^4^	0.023	3.13 × 10^6^	3.19 × 10^−7^	0.313	43
WT Ngb	2.50 × 10^8^	0.8	3.13 × 10^8^	3.20 × 10^−9^	6.50 × 10^7^	0.014	4.64 × 10^9^	2.15 × 10^−10^	14.9	38
Ngb-H64Q-CCC	7.20 × 10^8^	18	4.00 × 10^7^	2.5 × 10^−8^	1.60 × 10^8^	4.20 × 10^−4^	3.81 × 10^11^	2.63 × 10^−12^	9524	74
hemoCD-P	4.70 × 10^7^	1.30 × 10^3^	3.62 × 10^4^	2.77 × 10^−5^	1.30 × 10^7^	2.50 × 10^−4^	5.20 × 10^10^	1.92 × 10^−11^	1.44 × 10^6^	41, 89
RcoM-2	n.d.	n.d.	n.d.	n.d.	1.60 × 10^4^	6.40 × 10^−5^	2.50 × 10^8^	4.00 × 10^−9^	n.d.	98
RcoM-2 HBD	n.d.	n.d.	n.d.	n.d.	1.50 × 10^4^	3.50 × 10^−6^	4.29 × 10^9^	2.33 × 10^−10^	n.d.	98

Abbreviations: COX, cytochrome *c* oxidase; Hb-R, relaxed-state hemoglobin; Hb-T, tense-state hemoglobin; HBD, heme-binding domain; hemoCD-P, cyclodextran-based, small-molecule heme with pyridine linker; *K*_a_, association binding constant; *K*_d_, dissociation binding constant; *k*_off_, dissociation rate constant; *k*_on_, association rate constant; *M*-value, ratio of association binding constants for CO and O_2_ (K_a,CO_/K_a,O2_);Mb, myoglobin; Ngb-H64Q-CCC, an engineered variant of human neuroglobin; n.d., no data available; RcoM-2, regulator of CO metabolism, paralog 2 from *Paraburkholderia xenovorans*; WT, wild-type.
